# Differences in Hypotensive vs. Non-Hypotensive Sepsis Management in the Emergency Department: Door-to-Antibiotic Time Impact on Sepsis Survival

**DOI:** 10.3390/medsci6040091

**Published:** 2018-10-10

**Authors:** Leonor Ballester, Rafael Martínez, Juan Méndez, Gloria Miró, Manel Solsona, Elisabeth Palomera, Josep Anton Capdevila, Alejandro Rodriguez, Juan Carlos Yébenes

**Affiliations:** 1 Servei de Medicina Interna, Hospital de Mataró, Mataró, 08304 Barcelona, Spain; leoballesterj@yahoo.es (L.B.); jcapdevila@csdm.cat (J.A.C.); 2 Servei de Medicina Intensiva, Hospital de Mataró, Mataró, 08304 Barcelona, Spain; rmartinez@csdm.cat (R.M.); jmendezba@csdm.cat (J.M.); gmiro@csdm.cat (G.M.); msolsona@csdm.cat (M.S.); 3 Unitat de Recerca, Hospital de Mataró, Mataró, 08304 Barcelona, Spain; epalomera@csdm.cat; 4 Servei de Medicina Intensiva, Hospital Universitari de Tarragona Joan XXIII/IISPV/URV/CIBERes, 43005 Tarragona, Spain; ahr1161@yahoo.es

**Keywords:** sepsis, septic shock, antibiotic, mortality

## Abstract

Background: Sepsis diagnosis can be incorrectly associated with the presence of hypotension during an infection, so the detection and management of non-hypotensive sepsis can be delayed. We aimed to evaluate how the presence or absence of hypotension, on admission at the emergency department, affects the initial management and outcomes of patients with community-onset severe sepsis. Methods: Demographic, clinical, laboratory, process of care, and outcome variables were recorded for all patients, at the emergency department of our university hospital, who presented with community-onset severe sepsis, between 1 March and 31 August in three consecutive years. Patient management consisted of standardized bundled care with five measures: Detection, blood cultures and empirical antibiotics, oxygen supplementation and fluid resuscitation (if needed), clinical monitoring, and noradrenalin administration (if needed). We compared all variables between patients who had hypotension (mean arterial pressure <65 mmHg), on admission to the emergency department, and those who did not. Results: We identified 153 episodes (84 (54.5%) men; mean age 73.6 ± 1.2; mean Sequential Organ Failure Assessment (SOFA) score 4.9 ± 2.7, and 41.2% hospital mortality). Hypotension was present on admission to the emergency department in 57 patients (37.2%). Hemodynamic treatment was applied earlier in patients who presented hypotension initially. Antibiotics were administered 48 min later in non-hypotensive sepsis (*p* = 0.08). A higher proportion of patients without initial hypotension required admission to the intensive care unit (ICU) (43.1% for patients initially hypotensive vs. 56.9% in those initially non-hypotensive, *p* < 0.05). Initial hypotension was not associated with mortality. A delay in door-to-antibiotic administration time was associated with mortality [OR 1.150, 95%CI: 1.043–1.268). Conclusions: Initial management of patients with community-onset severe sepsis differed according to their clinical presentation. Initial hypotension was associated with early hemodynamic management and less ICU requirement. A non-significant delay was observed in the administration of antibiotics to initially non-hypotensive patients. The time of door-to-antibiotic administration was related to mortality.

## 1. Introduction

The incidence of sepsis [1] in developed countries is over 200 cases/100,000 inhabitants per year [2,3,4], and about 50 cases of community-onset sepsis/100,000 inhabitants/year require admission to intensive care units (ICU) [5]. The outcomes and the use of healthcare resources for community-onset sepsis are influenced both by factors that cannot be modified (e.g., infection site, causal microorganism, and patient characteristics) and by other, time-dependent, factors that can be modified, mainly early appropriate infection control and hemodynamic resuscitation [6]. Delayed or inappropriate resuscitation or infection-control measures are strongly associated with increased morbidity and mortality [7,8]. The heterogeneous clinical presentation of community-onset sepsis can make it difficult to detect, thus delaying the application of therapeutic measures and increasing mortality. We analyzed how the clinical presentation affects the management of patients with community-onset sepsis in our emergency department. Our main objective was to determine the effect of the absence of initial hypotension on emergency department management, and accordingly the impact on clinical evolution.

## 2. Materials and Methods

**Figure 1:**
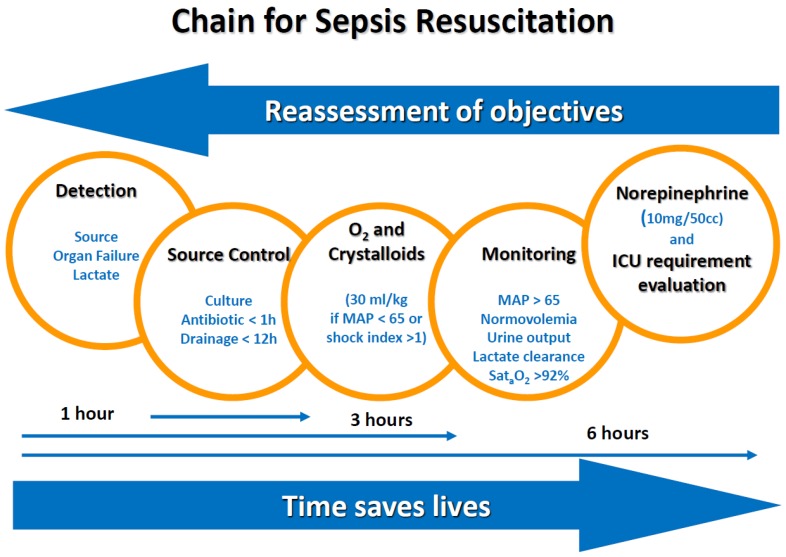
Chain for sepsis resuscitation that resumes the clinical algorithm for initial management of septic patients. ICU—intensive care unit; MAP—Mean Arterial Pressure.

This observational study collected data from all patients, at the emergency department of our university hospital, that fulfilled the sepsis-II criteria for severe sepsis [1], between 1 March and 31 August in three consecutive years (2008, 2009, and 2010). The study took place in a university hospital, with 320 acute beds and 14 ICU beds, that provides care for a population of 250,000 inhabitants. The management of patients was according to Surviving Sepsis Campaign recommendations [9]. A resuscitation chain for sepsis was developed by investigators [10] and was used to spread general recommendations for sepsis management (Figure 1).

The hospital’s clinical research ethics committee approved the study (serial number: 48/17) and waived the requirement for informed consent due to its observational nature. Patient information was appropriately encrypted before inclusion in the study database.

### 2.1. Patient Selection

We included all patients aged >18 years who presented, at the emergency department of our university hospital, with an acute community-onset infection, and who developed acute organ dysfunction or lactate concentrations above 2 mmol/L. We excluded patients undergoing palliative care, those with orders to withhold the life-support measures that were included in the initial resuscitation process (noradrenaline or mechanical ventilation), those who were diagnosed with severe sepsis and/or started resuscitation in another center, and those diagnosed with severe sepsis after transfer to hospital wards. 

We defined infection as a clinical process characterized by an inflammatory response to the presence of microorganisms; or the invasion of normally sterile host tissue by microorganisms in the absence of other causes for an inflammatory response. Systemic inflammatory response syndrome (SIRS) was defined if two or more of the following conditions were present: Temperature >38 °C, heart rate >90 beats per minute; respiratory rate >20 breaths per minute, and/or white blood cell count >12,000 or <4000. Sepsis was defined as an infection with the presence of two or more criteria of SIRS. Severe sepsis was defined as sepsis associated with organ dysfunction. Sepsis-induced hypotension was defined as mean arterial blood pressure <65mmHg, whereas septic shock was defined as maintained sepsis-induced hypotension despite adequate fluid resuscitation, which needs inotropic or vasopressor agents to restore perfusion abnormalities. To define organ failure, we used SOFA score criteria, requiring a score of ≥2 for at least one organ’s failure evaluation, being: Cardiovascular, respiratory, central nervous system, coagulation, liver or renal failure [11].

### 2.2. Data Collection

For patients presenting with severe sepsis in the emergency department, we recorded their demographics (age, sex, and comorbidity assessed with an age-adjusted Charlson Index) [12], clinical variables related to the presentation of sepsis (heart rate, blood pressure, respiratory rate, arterial oxygen saturation, and mental status), laboratory test results, focus of infection, and SOFA score [13]. We recorded the following process of care variables: Time from administrative admission to the obtainment of blood samples for cultures (door-to-culture time), to the administration of empirical antibiotics (door-to-antibiotic time), to the administration of 2 L of crystalloids (door-to-fluid resuscitation time), and to the administration of noradrenalin (door-to-noradrenalin time). We recorded the following outcome variables: Lactate levels 6 h after admission, the need for ICU admission, and hospital mortality. We compared variables between patients initially presenting with hypotension (mean arterial pressure (MAP) <65 mmHg) and those initially presenting without hypotension. Initial clinical variables were recorded during the first hour after administrative admission. The SOFA score was evaluated after 6h of admission. The time-to-procedure measurements were determined from the time of administrative admission to the time of the procedure.

### 2.3. Statistical Analysis

We used Student’s *t*-tests to compare continuous variables with normal distribution, Mann–Whitney U tests to compare continuous variables without normal distribution, and chi-square tests to compare categorical variables. We used simple logistic regression to calculate odds ratios and 95% confidence intervals. Statistical significance was set at *p* < 0.05. We used the forward stepwise method for the multivariate analysis.

## 3. Results

**Figure 2:**
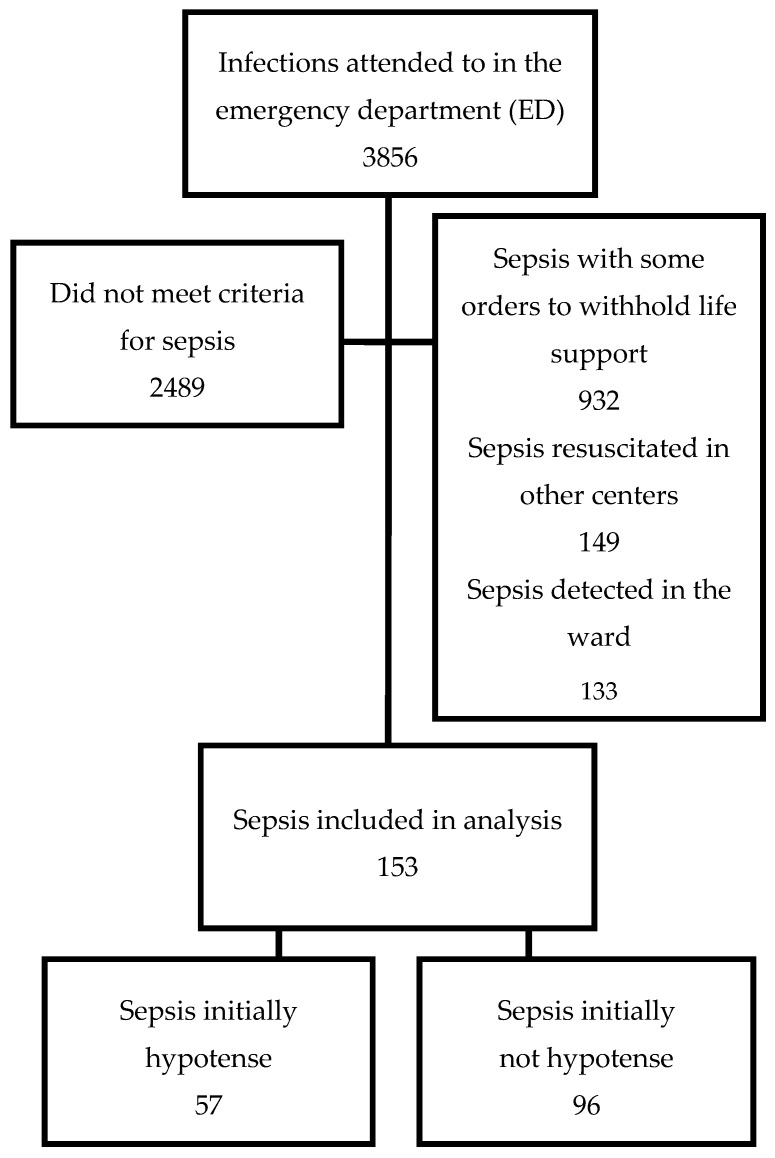
Enrollment flow chart.

During the 18 months comprising the study period, a total of 3856 patients were admitted to the hospital after presenting, at the emergency department, with acute community-onset infections; 321 (8.3% of the total) had acute organ dysfunction and/or elevated lactate concentrations, and 153 (4.0% of the total) of these were eligible for all resuscitation measures (Figure 2). 

Of the 153 patients included in the study, 84 (54.5%) were men, the mean age was 73.6 ± 1.2 years, and the mean age-adjusted Charlson score was 6.2 ± 2.9. The most common focus of infection was urinary (29.9%), followed by pulmonary (26%), abdominal (24%), and then unknown (13.6%). The mean SOFA score in the first six hours was 4.9 ± 2.7. Blood pressure, heart rate, and temperature were assessed in 100%, saturated O_2_ in 97.4%, and respiratory rate in 93%of patients. Mental status was considered normal if agitation, confusion, and coma were not reported (32%). Initial lactate concentrations were measured in 55 patients; the mean concentration was 5.2 ± 3.2 mmol/L. Hospital mortality was 41.2% (Table 1).

On arrival at the emergency department, 57 (37.2%) patients presented with initial hypotension. Hypotensive patients had higher initial lactate concentrations. Patients were managed differently according to whether they initially presented with hypotension or not (Table 2). Hypotensive patients received intervention measures earlier than those who were non-hypotensive; door-to-culture time, door-to-antibiotic time, door-to-fluid resuscitation time, and door-to-noradrenalin time were shorter in hypotensive patients. 

**Figure 3:**
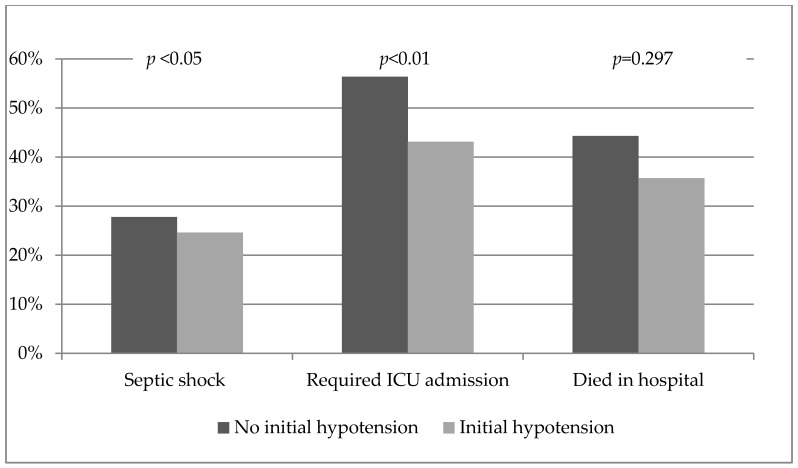
Differences in clinical evolution and outcomes between patients, with community-onset severe sepsis, that initially presented with hypotension and those that initially presented without hypotension.

Although the group of patients who were hypotensive at admission had higher initial lactate concentrations, lactate concentrations measured six hours after admission did not differ between the two groups. Fewer patients in the group with initial hypotension, compared to the group without initial hypotension, required admission to the ICU (43% vs. 56.9%, respectively, *p* < 0.01). No significant differences in hospital mortality were observed between the two groups (35.7% in the group with initial hypotension vs. 44.3% in the group without initial hypotension, *p* = 0.297) (Figure 3).

In the bivariate analysis, the initial presence of hypotension was not associated with mortality or the need for ICU admission. Initial symptoms associated with mortality were tachypnea, greater hypoxia, greater acidosis, higher lactate concentrations, and higher baseline blood glucose. Table 1 reports the differences between patients who survived the hospital stay and those who died in the hospital. 

The management of a patient during their stay in the emergency department had an effect on whether they required admission to the ICU and whether they survived the hospital stay, which correlated significantly with delayed administration of therapeutic measures. Patients who survived had shorter door-to-culture time, door-to-antibiotic time, door-to-fluid resuscitation time, and door-to-noradrenalin time than those who died in the hospital (Table 2). 

The multivariate analysis found that among the variables recorded in the emergency department, only age (OR 1.049; 95% CI: 1.020–1.079) and door-to-antibiotic time (OR 1.150; 95% CI: 1.043–1.268) were significantly associated with mortality. Retrospectively, a power analysis was performed. Accepting an alpha risk of 0.05 in a two-sided test, with 97 subjects in the no hypotension group and 57 in the hypotension group, the statistical power was 18% to recognize as statistically significant the observed differences in the mortality rates between groups. 

## 4. Discussion

We found that the time from admission to the administration of empirical antibiotics was delayed in patients with community-onset severe sepsis who were not hypotensive at the time of admission to the emergency department. In the multivariate analysis, time-to-antibiotic administration was the only modifiable factor associated with mortality. 

The main measures to prevent death in patients with severe community-acquired sepsis are controlling the focus of infection and restoring tissue perfusion. However, these apparently simple measures are difficult to implement. Only 19% of septic patients, from a worldwide multicenter study, received the complete management bundle within three hours of triage, which correlated with a lower hospital mortality rate when compared with septic patients that did not receive complete management within three hours of triage, (19.7% vs. 30.5%, respectively; *p* < 0.0001) [14]. These results show the need for strategies to improve the management of patients with sepsis [15,16,17].

Door-to-antibiotic time is considered one of the key elements in the management of community-onset severe sepsis [6,18,19,20,21,22,23]. Although it seems reasonable that patients who are hemodynamically unstable would undergo fluid resuscitation earlier than those who are not, it was surprising that the application of the infection-control measures in our study was also affected by patients’ hemodynamic presentation. The door-to-antibiotic time was shorter in patients with hypotension than in those without (3.6 ± 3.9 h vs.4.4 ± 4.2 h, respectively, *p* < 0.05), and this factor was independently associated with mortality. Other authors have documented how delays in identifying patients with severe sepsis are associated with delays in the administration of empirical antibiotics. In patients that presented, at the emergency department, with severe community-acquired pneumonia delays in pulseoximetry monitoring were associated with delays in door-to-antibiotic times, and consequently with worse survival rates [23].

In our study series, 37% of the patients with community-onset severe sepsis were hypotensive at triage, but only 25% required noradrenalin in the first six hours. Among our patients, initial presentation of hypotension was not a good indicator of mortality, probably because it was associated with earlier application of therapeutic measures. Thus, six hours after admission, lactate concentrations were similar among patients who initially presented hypotension and those who did not. The prognostic value of lactate concentrations measured six hours after admission in patients with severe sepsis, regardless of their blood pressure, has been widely reported [24].

Recently hypotension has been proposed as a major and easy clinical criterion to identify septic patients. Some authors are concerned about the possibility that it may delay the identification of septic patients in the early phase that could be called “pre-sepsis”. Moreover, several authors have found no differences in mortality between patients with hypotensive shock and those with cryptic shock (lactate >4mmol/L and tachycardia without hypotension) [25,26]. Thus, as our results suggest, it is important to detect sepsis or shock, regardless of the presence of hypotension [27,28,29,30]. In septic patients without comorbidities, tachycardia can raise blood pressure to normal levels through increased cardiac output, especially in children and younger adults in whom hypotension is a terminal phenomenon. To help in the detection of cryptic shock, the shock index can be useful, which is defined as the heart rate divided by the systolic blood pressure [31,32,33], or cutaneous refilling. Another useful measure is the use of sensors to detect somatic hypoxia-hypoperfusion (e.g., near-infrared spectrometry) in patients with normal blood pressure [34,35].

In our bivariate analyses, initial hypotension was not associated with mortality, but other factors were, for example, mortality was higher in patients who initially had hypoxia, tachypnea, or metabolic abnormalities (high lactate, hyperglycemia, and metabolic acidosis). These findings support the new definition of septic shock [36], which emphasizes the metabolic nature of shock beyond the purely macrohemodynamic characterization in earlier definitions. Systematic assessment of organ systems (respiratory system, circulatory system, central nervous system, etc.) has proven useful in detecting patients with life-threatening conditions. The qSOFA score has been proposed as an instrument to screen patients for sepsis [37]. Ot her, nonspecific mortality risk assessment scales include hypotension among other easily obtainable variables such as heart rate, respiratory rate, and oxygen saturation, and these can be easily applied in the emergency department. The modified early warning scale (MEWS) has been proven to be the most effective at identifying patients whose condition is likely to deteriorate [38], also specifically in septic patients [39].

The number of septic patients that were attended to at the emergency department, 1367 cases in 18 months, suggested an incidence of 365 cases/100,000 inhabitants; however, most of them had significant comorbidities and were finally excluded from the process of care analysis. The incidence of sepsis in this study was slightly higher than suggested by epidemiological local studies, based on ICD-9 or ICD-10 diagnosis codification system at hospital discharge [40]. Studies based on administrative codification can be useful to monitor trends of incidence or mortality, but they seem to underestimate real incidence, and therefore should be used with caution to provide an absolute value of incidence [41]. Although our study was not designed to analyze the incidence of sepsis, our results support this controversial topic.

The major limitations of our study were that it was carried out in a single center and included few patients, so caution is warranted when extrapolating our results. We observed a slight difference in antibiotic administration between both groups; however, it was not statistically significant because our study is clearly underpowered. A multicentric study, based on the monitoring of key process indicators has been presented to the Catalan Society of Intensive Care Medicine in order to be developed in the future. By not including patients whose resuscitation measures had started at other centers and patients with limits on life support (i.e., vasoactive drugs and/or mechanical ventilation), the size of our sample was restricted. Furthermore, because our aim was to analyze the effect of the presentation of vital signs at the emergency department on the outcomes in patients with community-onset severe sepsis, we calculated times to the application of therapeutic measures from nursing triage, rather than from the detection of sepsis (as specified in quality indicators).This therefore, made it difficult to compare our results with those of other studies. 

## 5. Conclusions

In conclusion, time from administrative admission to antibiotic administration was related to mortality in our study, so it could be used as a key process indicator of sepsis management in the emergency department. The presentation of hypotension on admission indicated more severe sepsis in patients, but as these patients were; accordingly, treated earlier than patients that did not initially present with hypotension, they consequently had fewer admissions to ICU. Our results underline the controversy about hypotension and sepsis detection. Relying excessively on the presentation of hypotension can lead to delays in hemodynamic management and the administration of antibiotics in septic patients with normal blood pressure, and thus affect prognosis. New studies are needed to confirm this hypothesis.

## Figures and Tables

**Table 1:** Comparison, between the survivors and non-survivors, among patients who presented with severe community-acquired sepsis at the emergency department.

	All Patients	Survivors	Non-Survivors	*p*	
	n = 153	n = 90 (59%)	n = 63 (41%)		OR (95% CI)
Gender					
Male	54.5%	62.2%	44.4%	0.03	2.06 (1.07–3.96)
Age					
Years	73.6 ± 15.78	70.3 ± 16.1	78.4 ± 14.2	0.003	1.04 (1.01–1.06)
Infection					
Urinary	29.9%	28.9%	30.2%	ns	0.93 (0.41–2.12)
Respiratory	26%	28.9%	22.2%	ns	1.42 (0.63–3.22)
Abdominal	24%	23.3%	25.4%	ns	0.94 (0.44–2.02)
Initial vital signs					
Presence of AhT	36.6%	40.0%	31.7%	ns	0.70 (0.35–1.37)
Systolic BP (mmHg)	106.2 ± 28.8	104.9 ± 30.4	108.1 ± 26.74	ns	1.00 (0.99–1.02)
MAP (mmHg)	75.5 ± 17.86	74 ± 20.18	77.5 ± 19.43	ns	1.01 (0.99–1.03)
HR (beats/minute)	99 ± 26.08	100 ± 24.03	98 ± 28.83	ns	0.99 (0.98–1.01)
RR (breaths/minute	25 ± 7.20	24.5 ± 6.50	26.9 ± 7.79	0.04	1.05 (0.99–1.10)
Sat O_2_ B (%)	91% ± 7.16	92.5 ± 6.67	89.8 ± 7.63	0.02	0.95 (0.91–0.99)
Glycemia (mg/dl)	154.2 ± 97.9	158.9 ± 19.7	191.7 ± 134.4	0.01	0.99 (0.99–1.00)
Temperature (°C)	37.06 ± 1.45	37.1 ± 1.5	37 ± 1.36	ns	0.97 (0.77–1.21)
Lactate					
0 h	5.24 ± 3.27	4.36 ± 2.1	6.68 ± 4.2	0.02	1.30 (1.04–0.62)
6 h	3.92 ± 3.35	3.00 ± 1.9	6.36 ± 5.03	0.001	1.38 (1.03–1.85)
SOFA score (6 h)	4.9 ± 2.74	4.83 ± 2.74	4.98 ± 2.73	ns	1.02 (0.91–1.15)
Time to measures					
Door-to-antibiotic	4.09 ± 4.13	3.38 ± 3.08 2.2 * (1.26–4.98) **	5.21 ± 5.20 3.25 * (1.46–7.20) **	0.05	1.12 (1.02–1.22)
Door-to-blood cultures	2.34 ± 3.24	2.21 ± 3.22	2.59 ± 3.29	ns	1.04 (0.93–1.16)
Door-to-fluid resuscitation	7.52 ± 4.28	7.14 ± 4.18	8.21 ± 4.42	ns	1.06 (0.96–1.17)
Required CVP	53 (34.9%)	33 (36.7%)	20 (31.7%)	ns	0.72 (0.36–1.42)
Door-to-CVP time	6.72 ± 4.18	5.74 ± 3.22	8.34 ± 5.08	0.06	1.18 (1.01–1.37)
Required noradrenalin	42 (27.5%)	25 (27.8%)	16 (25.4%)	ns	0.75 (0.36–1.55)
Door-to-noradrenalin time	7.59 ± 5.10	6.23 ± 3.65	9.72 ± 6.34	0.07	1.16 (1.01–1.34)

Continuous variables with normal distribution are presented as mean ± standard deviation. Door-to-antibiotic time include median * and interquartile range **. BP—blood pressure, MAP—mean arterial pressure, HR—heart rate, RR—respiratory rate, SatO_2_ B—basal oxygen arterial saturation, CVP—central venous pressure, AhT—Arterial hypotension, SOFA score—sequential organ failure assessment.

**Table 2:** Main epidemiological and clinical variables depending on the presence or absence of hypotension. Differences in response time (in hours), from the time of admission to the emergency department, for patients with initial hypotension versus those without.

	No Hypotension	Hypotension	*p*	
	n = 97 (63%)	n = 57 (37%)		OR (95%CI)
Gender				
Male	55.7%	52.6%	*ns*	1.13 (0.59–2.18)
Age				
Years	74.03 ± 15.94	72.86 ± 16.38	*ns*	0.99 (0.97–1.02)
Infection				
Urinary	24.7%	38.6	*ns*	0.52 (0.24–1.12)
Respiratory	26.8%	24.6%	*ns*	1.33 (0.59–3.05)
Abdominal	26.8%	19.3%	*ns*	1.81 (0.76–4.38)
Initial vital signs				
Systolic BP (mmHg)	121.48 ± 25.16	80.19 ± 9.37	*ns*	0.82 (0.76–0.88)
MAP (mmHg)	87 ± 19	55 ± 6.45		-
HR (beats/minute)	101.52 ± 25.58	96.17 ± 26.80	*ns*	0.99 (0.98–1.01)
RR (breaths/minute)	25.74 ± 6.92	25.28 ± 7.76	*ns*	0.99 (0.95–1.04)
Sat O_2_ B (%)	91.6 ± 7.17	90.96 ± 7.21	*ns*	0.99 (0.94–1.03)
Glycemia (mg/dl)	159.3 ± 85.0	145.8 ± 116.9	*0.06*	0.99 (0.99–1.00)
Temperature (°C)	37.16 ± 1.50	36.89 ± 1.36	*ns*	0.88 (0.69–1.10)
Lactate				
0 h	4.42 ± 2.22	6.23 ± 4.03	*0.03*	1.22 (0.99–1.50)
6 h	3.99 ± 3.50	3.75 ± 3.15	*ns*	0.98 (0.78–1.23)
SOFA score (6 h)	4.29 ± 2.41	5.95 ± 2.99	<0.001	1.26 (1.10–1.45)
Time to measures				
Door-to-antibiotic *	4.40 ± 4.25 2.63 * (1.62–6.32) **	3.62 ± 3.93 1.87 * (1.1–5.37) **	*0.08*	0.95 (0.87–1.04)
Door-to-blood cultures	2.68 ± 3.57	1.82 ± 2.59	*0.09*	0.91 (0.80–1.04)
Door-to-fluid resuscitation	8.49 ± 4.36	6.19 ± 3.83	*0.01*	0.87 (0.78–0.97)
Required CVP	33; 34.3%	20; 35%	*ns*	1.06 (0.53–2.12)
Door-to-CVP time	7.90 ± 4.15	4.78 ± 3.52	*0.004*	0.80 (0.67–0.95)
Required noradrenalin	27; 28.1%	14; 24.5%	*ns*	0.82 (0.39–1.74)
Door-to-noradrenalin time	8.99 ± 4.91	4.89 ± 4.46	*0.01*	0.81 (0.68–0.97)

Continuous variables with normal distribution are presented as mean ± standard deviation. Door-to-antibiotic time include median * and interquartile range **. BP—blood pressure, MAP—mean arterial pressure, HR—heart rate, RR—respiratory rate, SatO_2_ B—basal oxygen arterial saturation, and CVP—central venous pressure.
